# Mini review: Asymmetric Müllerian duct development in the chicken embryo

**DOI:** 10.3389/fcell.2024.1347711

**Published:** 2024-02-05

**Authors:** Juan L. Tan, Andrew T. Major, Craig A. Smith

**Affiliations:** Department of Anatomy and Developmental Biology, Monash Biomedicine Discovery Institute, Monash University, Clayton, VIC, Australia

**Keywords:** Müllerian duct, chicken sexual differentiation, AMH, Amhr2, oestrogen, sex determination, sexual differentiation

## Abstract

Müllerian ducts are paired embryonic tubes that give rise to the female reproductive tract. In humans, the Müllerian ducts differentiate into the Fallopian tubes, uterus and upper portion of the vagina. In birds and reptiles, the Müllerian ducts develop into homologous structures, the oviducts. The genetic and hormonal regulation of duct development is a model for understanding sexual differentiation. In males, the ducts typically undergo regression during embryonic life, under the influence of testis-derived Anti-Müllerian Hormone, AMH. In females, a lack of AMH during embryogenesis allows the ducts to differentiate into the female reproductive tract. In the chicken embryo, a long-standing model for development and sexual differentiation, Müllerian duct development in females in asymmetric. Only the left duct forms an oviduct, coincident with ovary formation only on the left side of the body. The right duct, together with the right gonad, becomes vestigial. The mechanism of this avian asymmetry has never been fully resolved, but is thought to involve local interplay between AMH and sex steroid hormones. This mini-review re-visits the topic, highlighting questions in the field and proposing a testable model for asymmetric duct development. We argue that current molecular and imaging techniques will shed new light on this curious asymmetry. Information on asymmetric duct development in the chicken model will inform our understanding of sexual differentiation in vertebrates more broadly.

## 1 Introduction

Morphogenesis of the female reproductive tract is critical for sexual reproduction. Understanding how the female reproductive tract develops provides important information on a critical organ system and can also broadly inform other areas biology. The female reproductive tract of amniotic vertebrates (reptiles, birds and mammals) derives from a pair of embryonic tubes called the Müllerian ducts. Early embryos of both sexes develop two pairs of undifferentiated ducts is close association with the mesonephric kidneys and the gonads; these are the Wolffian and Müllerian ducts. In male mammals, under the influence of testis-derived androgens, the Wolffian ducts become the vas deferens and epididymis of the male reproductive tract, while the Müllerian ducts disintegrate under the influence of the testis-derived factor, Anti-Müllerian Hormone (AMH). Conversely, in females, the Wolffian ducts regress and the absence of AMH allows differentiation of the Müllerian ducts into the oviduct (in mouse and chicken) and homologous structures in humans: Fallopian tubes, uterus and upper vagina) (Wilson, 1978; van Tienhoven, 1983; Cate, 2022; Machado et al., 2022). Hence, proper sexual differentiation of the Müllerian ducts during embryogenesis is central to female reproductive tract development. Formation of these structures also provides a model for understanding how tubes form in biological systems more broadly (tubulogenesis).

## 2 AMH and sexual differentiation of the Müllerian ducts

**FIGURE 1:**
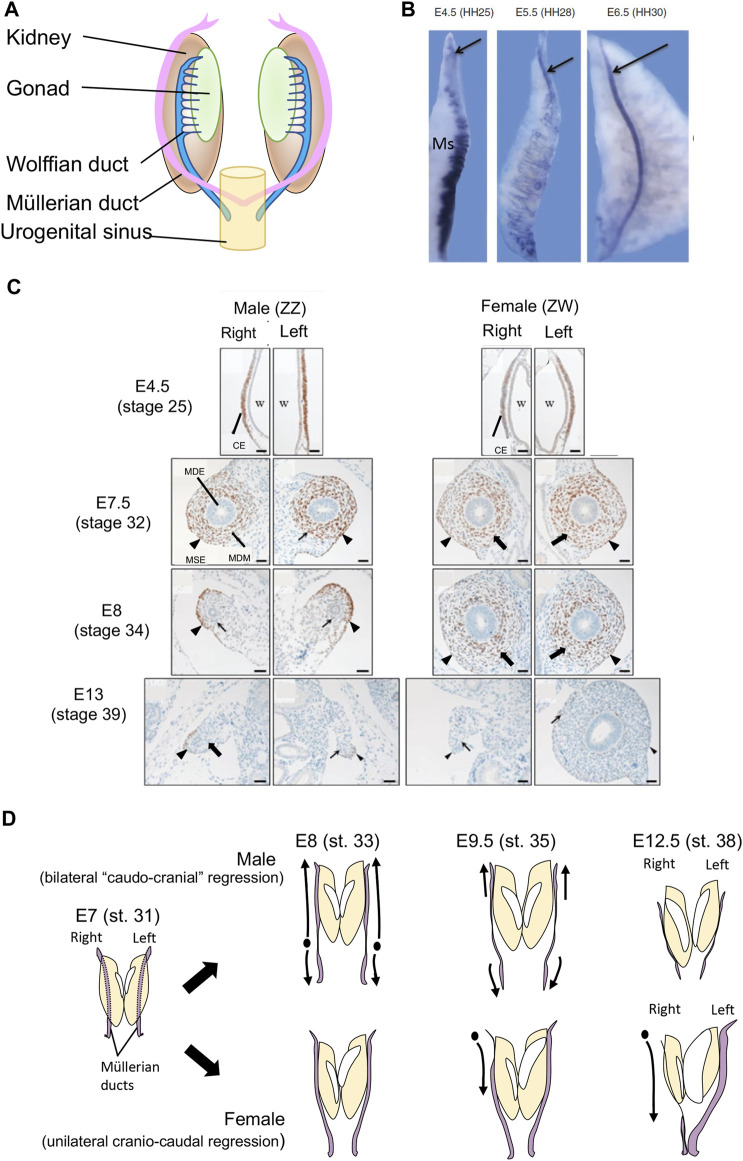
**(A)** Schematic of Müllerian ducts in amniotic vertebrate embryos. Müllerian ducts form in close association with the Wolffian ducts, on the surface of the embryonic kidneys (mesonephric kidneys). **(B)** Formation of the Müllerian duct in the chicken embryo, based on expression of the marker, *LIM1* (arrow). At E4.,5 (HH stage 25) the duct anlagen appears as a group of LIM1+ cells at the cranial pole of the mesonephric kidney (Ms), which also expresses some Lim1 in its tubules. AT E5.5 (HH stage 28), the duct elongates caudally through cell proliferation and caudal expansion. By E6.5 (HH stage 6.5) the duct has migrates to the posterior end of the urogenital system. **(C)** Transverse histological sections showing chicken Müllerian duct development, based on immunohistochemical staining for the marker protein, DMRT1. The ducts first form as DMRT^+^ thickenings of coelomic epithelium (CE) overlying the Wolffian duct (W) at E4.5 (stage 25). In both the left and right sides of both sexes, this thickening gives rise to cells that migrate to form the inner Müllerian duct epithelium (MDE) and surrounding Müllerian duct mesenchyme (MDM) by E7.5 (stage 32). Both the Müllerian surface epithelium (MSE; arrowhead) and the MDM (arrow) express DMRT1. At E8 (stage 34) both male ducts undergo regression, marked by diminished mesenchyme (arrowhead) and smaller MDE (arrow). At E13 (stage 39), both male ducts are regressed and no longer express DMRT1. In females, the right duct has also regressed (arrowhead and arrow), while the left duct is enlarged. It has an expanded mesenchymal domain and duct lumen, and no longer expresses DMRT1. Modified from Omotehara et al. (2014), with permission. **(D)** Schematic of chicken Müllerian duct development and regression in male and female embryos. In males, bilateral duct regression commenced from E7-E8 (stage 31–33). Regression of both ducts occur simultaneously and largely progresses in a caudo-cranial direction. In females, right duct regression commences slightly later, from E9.5 (stage 35) and progressed in a cranio-caudal direction. From the same stage, the left female duct increases in size. By E12.5, both male ducts and the right female duct have regressed, while the left female duct is well developed.

In vertebrate embryos, the Müllerian ducts initially form in both sexes as tubes that run from the cranial to caudal pole on either side of the embryonic kidneys (mesonephric kidneys) (Figure 1A). The ducts derive from a thickened placode of cells in the coelomic epithelium overlying the cranial pole of the mesonephros. This placode comprises Müllerian epithelial and mesenchymal progenitor cells. The cells proliferate and invaginate, giving rise to a meso-epithelial tube (the Müllerian epithelium) and surrounding mesenchyme (Jacob et al., 1999; Guioli et al., 2007; Orvis and Behringer, 2007). As development proceeds, the Müllerian epithelium elongates by caudal extension through the mesenchyme, until it reaches the urogenital sinus reviewed in (Klattig and Englert, 2007; Santana Gonzalez et al., 2021). This is a conserved process among amniotic vertebrates, exemplified by the chicken embryo (Figure 1B). In mouse and/or chicken models, several genes and signalling pathways have been identified that regulate early duct specification, invagination and elongation, including the transcription factors, Lim1, Pax2, Emx2 and Dach1/2, together with Fgf, Bmp and Wnt4 signaling (Miyamoto et al., 1997; Bouchard et al., 2002; Kobayashi et al., 2004; Biason-Lauber and Konrad, 2008; Davis et al., 2008; Atsuta and Takahashi, 2016; Prunskaite-Hyyrylainen et al., 2016). Genetic manipulation of these factors blocks or impairs Müllerian duct formation (Torres et al., 1995; Vainio et al., 1999; Kobayashi et al., 2004; Orvis and Behringer, 2007; Huang et al., 2014). By embryonic day (E)13.5 in mouse and E6.5 in chicken, the ducts are well formed in both sexes. Subsequently, the fate of the Müllerian ducts in males and females diverges dramatically. Figure 1C shows Müllerian duct formation and sexual differentiation in the chicken embryo, based on transverse histological sections stained for the marker, DMRT1. As in mouse, the Müllerian duct anlagen in chicken appears as a DMRT + thickening of coelomic epithelium adjacent to the Wolffian duct. This occurs as embryonic day (E)4.5. By E7.5, proliferation and inward migration of coelomic epithelial cells has given rise to centrally located Müllerian epithelium surrounded by DMRT1+ mesenchyme. Bilateral duct regression in male embryos commences from E7.5-8 (stage 33–34). By E13 (stage 39) both male ducts are largely completely regressed (Figure 1C). In most birds, only the left duct develops into an oviduct (Figure 1D). The right Müllerian duct regresses in females, commencing from E9.5, slightly later than in male embryos (Hutson et al., 1983). This is in parallel with regression of the right gonad, which becomes vestigial. (Figures 1C, D).

In mammals, regression of the Müllerian ducts occurs in male but not female embryos under the direction of Anti-Müllerian Hormone (AMH, also called Müllerian Inhibiting Substance, MIS) (Josso and Picard, 1986; Behringer et al., 1990; Josso et al., 1993a; Josso et al., 1993b; Behringer, 1994; Behringer et al., 1994). In the mammalian embryo, AMH expression is first detectable in developing Sertoli cells of the nascent testis, regulated by factors such as Sox9, Wt1 and Sf1 (De Santa Barbara et al., 1998). In the mouse model, the *Amh* gene is expressed from E12.5, soon after the onset of the master testis-determinant, *Sry*, and pre-Sertoli cell differentiation (Munsterberg and Lovellbadge, 1991). *Amh* is not expressed in the female mouse embryos. Müllerian duct regression commences in the male mouse embryo from E13.5. It is assumed that Amh enters the embryonic blood stream from the nascent testes to exert its effects upon the adjacent Müllerian ducts, although diffusion from the gonads through the mesonephros to the duct is also possible. To induce regression, Amh must bind its cognate receptor, Amhr2, which recruits type I receptor (Josso et al., 1998; Mishina et al., 1999). These TGF-β receptors are serine-threonine kinase receptors that engage intracellular SMAD signalling to induce duct regression in males. In mouse, it has been shown that activation of Amhr2 recruits either Bmpr1a (Alk3) or Acvr1 (Alk2) type I receptors, and the three BMP receptor-Smads (Smad1, Smad5, and Smad8) function redundantly in transducing the Amh signal required for Müllerian duct regression (Visser et al., 2001; Jamin et al., 2002; 2003; Orvis et al., 2008). In mammals, the *Amhr2* gene is expressed in duct mesenchymal cells of both sexes from E12.5, based on LacZ reporter studies. However, only male embryos produce Amh, from E12.5, resulting in male-specific bilateral duct regression commencing from E13.5-E14.5 (Arango et al., 2008). *Amh*-induced regression involves activation of Wnt signaling through β-catenin, expression of metalloproteases and apoptosis (Moses and Behringer, 2019) reviewed in (Klattig and Englert, 2007; Mullen and Behringer, 2014).

## 3 AMH and Müllerian duct regression in the chicken embryo

**FIGURE 2:**
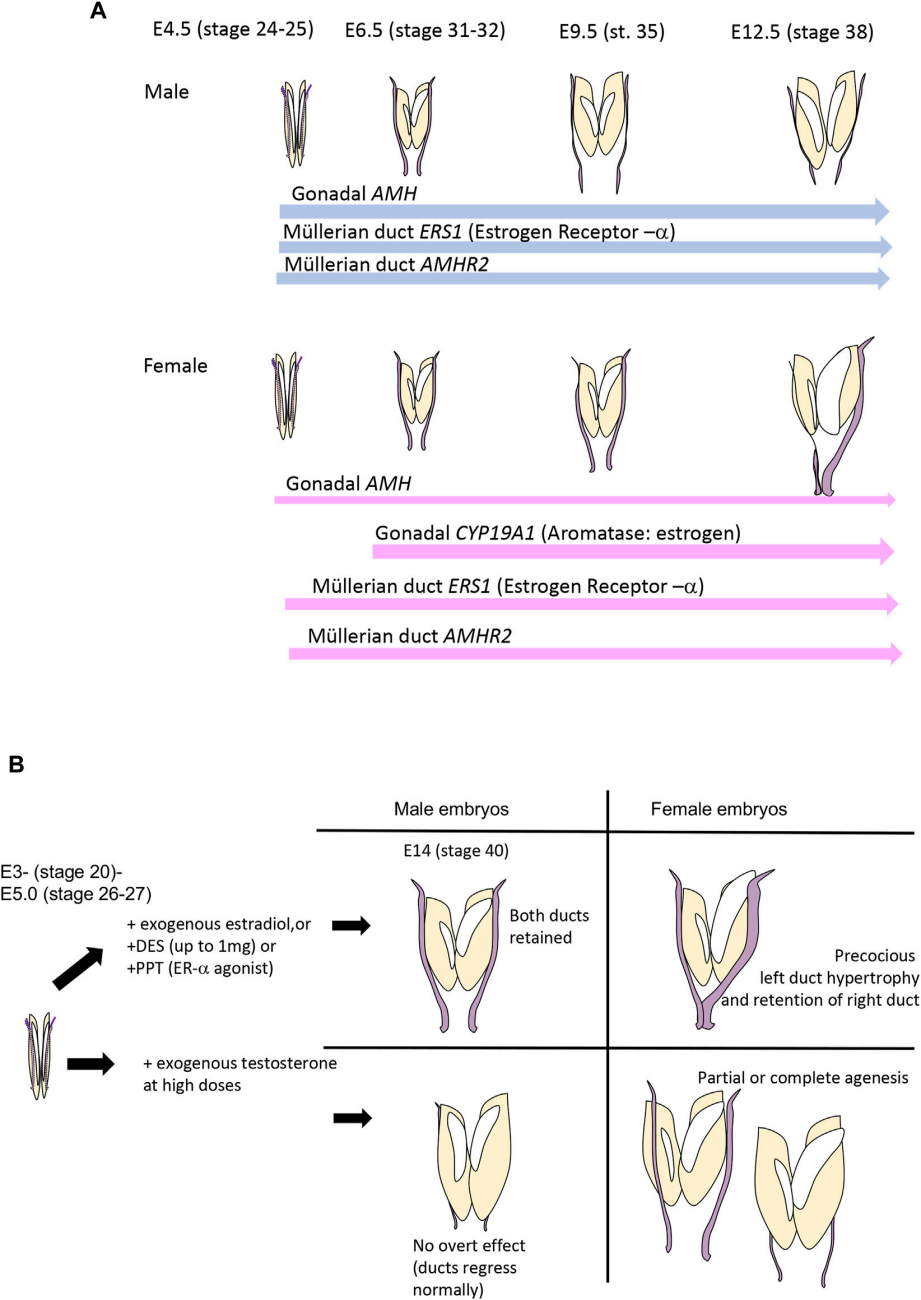
**(A)** Timeline of critical gene expression relevant to Müllerian duct regression in the chicken embryo. AMH is expressed in the gonads of both sexes at E4.5 (stage 25) even before the onset of gonadal sex differentiation at E6.0-E6.5. Expression is always higher in males. Expression of *AMHR2* occurs in the ducts of both sexes, and in both sides, from E4.5. Precise quantification has not been reported. *ESR1*, encoding estrogen receptor-α, is also expressed in both ducts of both sexes, from E4.5, but, again, precise quantification has not been reported. Estrogen is only produced in female embryos, as *CYP19A1*, encoding Aromatase enzyme, is only expressed in (both) female gonads from E6.0—E6.5 (HH stage 31–32). Data from Smith et al. (1997) and Cutting et al. (2014). **(B)** Effects of sex steroid hormones, their agonists and antagonists on avian Müllerian ducts. Estrogens have a stimulatory effect and testosterone (at high doses) has an inhibitory effect.

The chicken embryos exhibits an unusual pattern of duct regression that serves to broaden our understanding of female reproductive tract formation. The Müllerian ducts form in the chicken embryo in the same way as in mammals, involving specification, invagination and elongation (Guioli et al., 2007; Atsuta and Takahashi, 2016; Roly et al., 2018; Roly et al., 2020b). The Müllerian duct of birds, like that of mammals, becomes regionally differentiated after hatching. In birds, it gives rise to the oviduct that has specialised shell gland, isthmus and other compartments (Bakst, 1998) reviewed in (Major et al., 2022). While both ducts regress in male chicken embryos, the right duct also regresses in females, accompanying regression of the right gonad. Why the right female gonad and its associated duct regress is not entirely clear. It has been hypothesised that such unilateral regression of the right duct and gonad makes birds lighter, an energetic advantage for flight, or that bilateral gravid ovaries and ducts would cause mechanical damage to developing eggs [reviewed in (Guioli et al., 2014)]. Gonadectomy in early chicken embryos results in the retention of both ducts in both sexes, showing that intact gonads are required for duct regression (Hutson et al., 1983). The gonads must secrete hormones that regulate duct regression–bilaterally in males and asymmetrically in females. As in mammals, AMH is expressed in the embryonic gonads of male chicken embryos from the onset of gonadal sex differentiation, from E4.5, equivalent to Hamilton-Hamburger (HH) stage 21 (Eusebe et al., 1996; Oreal et al., 1998) (Figure 2A). AMH, or an implanted testis secreting AMH or viral vector over-expressing AMH, can induce Müllerian duct regression in chicken embryos, as in mammals (Maraud et al., 1982; Rashedi et al., 1983; Lambeth et al., 2016). However, unlike in mammals, the female avian gonad also produces AMH at embryonic stages. In the chicken embryo, AMH expression in female embryonic gonads commences at the same time as in males, but at a lower level (Hutson et al., 1981) (Figure 2A) Based on immunostaining and organ culture assays, it has been shown that the left female chicken gonad produces a higher level of AMH than the right gonad, which ultimately loses the ability to secrete AMH (Hutson et al., 1981; Oreal et al., 1998). AMH expression female gonads provides the mechanism of right duct regression in that sex. The higher level of AMH activity in left versus right female gonads most likely reflects the larger size of the left gonad, and regression of the right gonad. Higher levels of AMH in the left versus right female gonad are unlikely to be the mechanism underpinning right duct regression, as right duct regression occurs despite lower levels of gonadal AMH in the right gonad. Hence, while the left female gonad produces more AMH then the right, it must enter the bloodstream to induce regression of the right (contralateral) duct.

Several studies have shown that left-right asymmetry of the avian gonad is mediated by the *PITX2* transcription factor, which regulates a molecular cascade that triggers enhanced cell proliferation in the left gonad (Guioli and Lovell-Badge, 2007; Ishimaru et al., 2008; Rodriguez-Leon et al., 2008). This molecular pathway contributes to the smaller size of the right female gonad, and hence a lower level of AMH output. However, there is no evidence to suggest that such a mechanism also underpins the left-right asymmetry of the female Müllerian ducts. *PITX2* is not expressed in the ducts and, indeed, both left and right ducts initially develop in both sexes. Rather, the asymmetric fate of the left versus right female Müllerian ducts is hormonally driven, based on the endocrine output of the gonads.

Early anatomical studies conducted on chicken and Pekin duck embryos showed that the right duct of female embryos regresses in a different manner to that of the bilateral regression in males. In the female, the right duct regresses cranio-caudally (i.e., disintegration starts at the anterior pole and progresses down) (Figure 1D) (Lillie, 1919; Lewis, 1946). In males, regression commences slightly earlier and first occurs in the lower medial half of the duct, then largely progresses cranially (so-called “caudo-cranial” regression) (Figure 1D) (Romanoff, 1960; Groenendijk-Huijbers, 1962). Furthermore, bilateral regression is rapid in males, spanning days 8–10, while it is more gradual in females, from E9.5 through E16 (Hutson et al., 1983). These differences between the sexes in the directionality of regression are intriguing and have not been explained at a molecular level. The left female duct of female chicken embryos continues to grow in length and thickness through days 10–21, during which time it enlarges caudally to form the future shell gland (Hutson et al., 1983).

In the chicken embryo, *AMHR2* is expressed in the Müllerian ducts and gonads of both sexes from early stages, prior to subsequent duct regression (Cutting et al., 2014). (Figure 2A). Expression of AMH by female gonads provides the mechanism of duct regression in that sex. However, no studies have been conducted to suggest that different sensitivity to AMH or level of AMHR2 expression could explain the sex differences in the directionality of duct regression shown in Figure 1D. However, in females, the question emerges as to how the left duct is protected from the regressive effects of AMH, despite expressing both AMH from the gonad and AMHR2 in the duct. Several lines of evidence indicate a protective effect of estrogen, which is produced by the gonads at high levels only in females (Figure 2A).

## 4 Sex steroid hormones and asymmetric chicken Müllerian duct development

Sex steroid hormones do not play an overt role in Müllerian duct formation or regression in mammals. However, several lines of evidence invoke sex steroids, primarily estrogen, in mediating the left-right asymmetry of chicken Müllerian duct development. The effects of sex steroid hormones, their analogues and antagonists on chicken Müllerian ducts, are shown in Figure 2B. Given that both sexes of chicken embryos produce AMH, and the right female duct regresses due to AMH (or a surgically implanted testis) (Stoll et al., 1975; Maraud et al., 1982; Hutson et al., 1983), the question arises as to why the left female duct does not also regress? Several lines of evidence invoke a protective effect of estrogen, which is synthesised by female but not male embryonic chicken gonads from E6 (stage 29) (Smith et al., 1997; Nomura et al., 1999; Smith and Sinclair, 2004; Hirst et al., 2018) (Figure 2A). Exposure of male avian embryos to exogenous 17β-estradiol or estrogen analogues such as increasing doses of diethylstilbestrol (DES) at early stages completely prevents bilateral duct regression (Hutson et al., 1982; Hutson et al., 1985; Doi and Hutson, 1988; Stoll et al., 1993) (Figure 2B). Administration of ER-α agonists has a similar effect (Mattsson et al., 2011)This is consistent with the known expression of estrogen receptor-α in male (and female) ducts (Andrews et al., 1997). In females, early exposure to exogenous estrogen at E2-3 causes precocious differentiation of the left duct, characterised by thickened epithelia and mesenchyme and the appearance of tubular glands (Andrews and Teng, 1979) (Figure 1B). This indicates that estrogen plays a role in left female duct differentiation. The striking effects of estrogen administration to male embryos indicates that estrogen in some way antagonises the action of AMH in directing duct regression, and would be the putative mechanism by which the left female duct is retained.

The mechanism by which estrogen antagonsies AMH function in the context of asymmetric Müllerian duct regression has been controversial. This antagonism may be in the gonad, where estrogen may repress AMH gene expression or function, or at the level of the duct, where estrogen may block AMH action. In the chicken, implantation of a day 13 testis into the coelom of an early (day 3) female embryo causes complete regression of both Müllerian ducts (Maraud et al., 1982). This firstly confirms that AMHR2 is expressed in both female ducts (as in males) but it also shows that high levels of AMH from the older stage testis can direct duct regression if it pre-empts estrogen action, (Stoll et al., 1987). Stoll and colleagues argue that the role of estrogen in females is downregulation of AMH secretion by the gonads, based on earlier observations that the estrogen-induced maintenance of ducts in males is blocked by the potent estrogen antagonist, tamoxifen, but tamoxifen treatment alone cannot cause duct regression in females (as might be expected if estrogen acted protectively at the level of the duct) (Stoll et al., 1993). One issue with this interpretation is that, in some contexts, tamoxifen may act as an estrogen agonist, not an antagonist, as can occur, for example, in reptile embryos (Lance and Bogart, 1991). The alternative view, which has more experimental support, is that estrogen acts to antagonise AMH function at the level of the duct itself. Using *in vitro* organ culture experiments, Hutson, Doi and colleagues found that pre-treatment of male chicken embryos with the estrogen analogue, DES, protects ducts for regression *in vitro* when exposed to an older stage testis secreting AMH. (Hutson et al., 1982; Doi and Hutson, 1988). This suggests that duct retention is not caused by suppression of AMH secretion by DES, but by direct antagonism of AMH action in the ducts themselves. The molecular details of this interaction are currently unknown, but would provide insight into the interaction between sex steroid and AMH function more broadly. Activated estrogen receptor may directly downregulate *AMHR2* gene expression in the left female duct, for example, although evidence for this is lacking. Both left and right males and female ducts appear to express *AMHR2* throughout embryogenesis (Cutting et al., 2014). Alternatively, ERα may act in competition with the AMHR2 effectors, SMADs, directly at target genes involved in duct regression (e.g., apoptosis and matrix metalloproteases).

The next question that arises is why estrogen does not also protect the right duct from regression in females, as it does in the left? A logical possibility would be a lack of estrogen receptor expression the right duct. We and others have shown that *ERα* mRNA expression or E2 binding occurs in both left and right Müllerian ducts in both sexes of chicken embryos, at least up to E7.5 (Hutson et al., 1983; Andrews et al., 1997). ER-β is not expressed in the ducts (Mattsson et al., 2011). It is possible that ER-*α* expression becomes asymmetric beyond E7.5. The presence of estrogen receptors in chicken Müllerian ducts has been inferred from studies conducted in the 1970’s and 1980’s using radio-labelled estradiol binding assays. Using this approach, it has been reported that ER is expressed in the left female Müllerian duct from E8 (Teng and Teng, 1975). Furthermore, one study published in 1983 used ^3^H-estradiol binding to show an apparent difference in E2 binding between left and right embryonic chicken Müllerian ducts, where binding was higher in the left compared to the right ducts (MacLaughlin et al., 1983). This may explain the differential actions of E2 on the left versus right ducts in females. However, an independent study found no difference in the biochemical characteristics of E2 binding in left vs. right ducts (Reichhart et al., 1980). Modern molecular tools need to be applied to answer this question. The other converse possibility is that AMHR2 protein (or the type I receptor) is not expressed in the left female duct, although we do note *AMHR2* mRNA expression both left and right female (and male) ducts over development in the chicken embryo (Cutting et al., 2014). Furthermore, type I receptors (*ACVR1* and *BMPR1a*) are expressed in chicken embryonic ducts, at least at early stages (Roly et al., 2020a). However relative quantification of type I and type II proteins in male vs. female and in left vs. right ducts has never been assayed.

In chicken, it has been shown that the embryonic gonads of both sexes produce measurable amounts of 17β-estradiol and testosterone during the period of Müllerian duct sexual differentiation (from E7-8 onwards). The early synthesis of estrogen by female gonads has been well documented (Woods and Erton, 1978; Woods and Brazzill, 1981). Androgens are synthesised in the gonads of both sexes and could, in theory, influence duct development (Woods and Podczaski, 1974; Woods et al., 1975). When very high doses of exogenous testosterone are administered to female chicken embryos *in vitro* or *in ovo*, the Müllerian ducts can disintegrate, but some other studies found no effect or partial effects at lower doses [reviewed in (Hutson et al., 1983)] (Figure 2A). This most likely reflects the timing, dosage and route of administration of testosterone. It has been considered that many studies examining the effects of testosterone have used pharmacological doses. At physiological doses, testosterone appears to augment AMH-induced duct regression (Ikawa et al., 1982). Androgen receptor is expressed in chicken Müllerian duct (Reichhart et al., 1980; Katoh et al., 2006). In the chicken embryo, Autoradiography and gene expression analysis indicate that AR is expressed in the mesenchyme from the early stages of duct formation (E5.5- E6.5) through regression and differentiation (E10.5+) (Gasc, 1981).

## 5 Answering questions of Müllerian duct development with modern molecular and imaging approaches

The studies on avian Müllerian duct development described above have largely involved gross anatomy, histology and approaches such radio-labelled sex hormone binding, conducted in the last century. Today, we have access to genomic and transcriptomic tools and advanced imaging methods that will shed new light on duct formation and, especially, the molecular basis of the curious asymmetry seen in most birds. These tools will be very useful in teasing out the hormonal interplay that underpins asymmetric duct development in the avian model. This, in turn, will provide further insights into duct formation and sexual differentiation in vertebrates more broadly. For example, advances in whole mount immunofluorescent staining of ducts allows the visualisation and quantification of duct development and regression on a global level, using methods such as multi-plane whole mount confocal imaging. Such approaches can now be applied to the study of ERα and AMHR2 expression along the entire duct, to discern the mechanism of asymmetry in females (different patterns of ERα or different AMHR2 protein expression?) and the cranio-caudal (female) vs. caudo-cranial (male) regression profiles. Single cell RNA-seq and ATAC-seq studies can also be performed to elucidate the different cell populations and global gene regulatory landscape of duct development.

Comparisons can be drawn between natural Müllerian duct asymmetry in birds and atypical duct formation among mammals. Such comparisons highlight shared and diverged molecular mechanisms. In the chicken, for example, AMH levels are higher in the left gonad than in the right, yet it is the right duct that regresses. This is presumably because AMH enters the bloodstream or diffuses to the ducts regardless of its site of origin (where the left duct is protected via secreted estrogen). In mice, unusal unilateral Müllerian duct regression has been reported in mutants such as B6N-XY^POS^ (retarded *Sry* expression) and in *M33* and *jumonji domain-containing protein 1a* (Jmjd1a)-knockout mice (Katoh-Fukui et al., 1998; Kuroki et al., 2013; Yamamoto et al., 2018). In these mice, a percentage develop as, true hermaphrodites, characterised in which the left gonad is an ovary, with neighbouring left Müllerian duct, and the right gonad is a testis and the right duct regresses. At least in the case of the B6N-XY^POS^ mice, has been shown that this asymmetry involves local diffusion of AMH on the right side of the urogenital system only (Yamamoto et al., 2018). Such ipsilateral effects of AMH are also evident in rabbit embryos in which the testis is removed from one side: the Mullerian duct on the operated side is maintained (Jost, 1953). This is different to the contralateral mechanism inferred in birds. In human females, one Mullerian duct can sometime fail to develop, leading to a so-called unicornate uterus (a small uterus with only one Fallopian tube). While the incidence of unicornate uterus is sporadic and rare (0.3% of the population), the cause is unknown (Trad and Palmer, 2013). Studies on the asymmetric sensitivity of the paired Müllerian ducts to AMH or oestrogen in chicken may shed light on asymmetric defects in human Müllerian duct formation.

## 6 Conclusion

In mammals, it is the presence or absence of AMH that underpins sexually dimorphic fate of the Müllerian ducts. In the chicken embryo, the picture is more complex, with asymmetric duct regression occurring in females. This pattern can shed light on the molecular mechanisms of duct regression more broadly among animals, as outlined above. The chicken model offers several novel perspectives on the roles of AMH and sex steroid hormones in the vertebrate reproductive tract. Firstly, during embryonic life, the ovary in addition to the testis produces AMH. Secondly, the female right duct regresses as do both males ducts. Thirdly, the pattern of regression differs between the sexes (cranio-caudal in females vs. largely caudo-cranial in males). These features make the chicken embryo an attractive model for studying genetic and hormonal regulation of the developing Müllerian ducts. In the chicken model, duct development can now be readily manipulated *in ovo*, using modern molecular methods such as electroporation of genes for over-expression, or shRNAs for gene knockdown or Cas9/guide RNAs for gene knockout (Guioli et al., 2007; Atsuta and Takahashi, 2016; Roly et al., 2020b). These rapid functional approaches will provide new information on how the Müllerian ducts grow and undergo sexual differentiation. Furthermore, studies on the hormonal regulation of Müllerian duct formation in egg-laying species such as the chicken are be important for understanding the effects of teratogens and xenoestrogens on sexual differentiation (Mattsson et al., 2011).
